# Complete laparoscopic-transhiatal removal of duplex benign oesophageal tumour: case report and review of literature

**DOI:** 10.1186/s12876-021-01625-8

**Published:** 2021-02-02

**Authors:** László Andrási, Zoltán Szepes, László Tiszlavicz, György Lázár, Attila Paszt

**Affiliations:** 1 Department of Surgery, University of Szeged, Semmelweis u. 8, 6725 Szeged, Hungary; 2 1St Department of Internal Medicine, University of Szeged, Korányi fasor 8-10, 6720 Szeged, Hungary; 3 Department of Pathology, University of Szeged, Állomás u. 2, 6725 Szeged, Hungary

**Keywords:** Laparoscopic-transhiatal, Duplex oesophageal leiomyoma, Case report

## Abstract

**Background:**

Leiomyoma is the most common benign oesophageal tumour. Half of all leiomyoma patients have oesophagus-associated complaints, such as dysphagia and epigastric pain, and the other 50% are asymptomatic with a diagnosis made on incidental discovery. Endoscopic ultrasonography is essential for an accurate preoperative workup and can enable guided-tissue acquisition for immunohistochemistry in certain cases. Smaller tumours are amenable to traditional and novel endoscopic removal in specialized centres, but some complex cases require surgical enucleation with a minimally invasive approach.

**Case presentation:**

An asymptomatic 60-year-old woman was accidentally diagnosed with a bifocal oesophageal mass, which was discovered by chest computed tomography. We report a rare case of a duplicated lower-third oesophageal leiomyoma, which was completely removed via the laparoscopic transhiatal approach. The patient has recovered successfully from the surgery. She has been followed up for six months with a normal oesophagram, adequate oesophageal function and no complaints observed. Pathological examination confirmed the diagnosis of leiomyoma in both lesions.

**Conclusions:**

To the best of our knowledge, this is the first reported case of duplex oesophageal leiomyomas removed laparoscopically. Using the minimally invasive abdominal technique, the lower oesophagus can be mobilised to the mediastinum without pleura injury and offers a good alternative to the thoracoscopic approach in patients with possible intrathoracic difficulties. At experienced centres, laparoscopic transhiatal enucleation of lower oesophageal leiomyomas and other benign tumours with a combination of intraoperative oesophagoscopy is a safe, fast and effective operation.

## Background

Benign oesophageal tumours are rare, with a prevalence of less than 0.5% [1], while benign tumours represent 20% of oesophageal neoplasms on autopsy [2]. Besides stromal tumours, granular cell tumours, lipomas and schwannomas, oesophageal leiomyoma is one of the most common types of benign oesophageal tumours, accounting for 80% of nonmalignant cases with male dominance [3, 4]. Approximately 50% of patients are asymptomatic with a diagnosis made on incidental discovery. When symptomatic, symptoms include dysphagia, retrosternal pain, heartburn, regurgitation or occasionally upper gastrointestinal bleeding and weight loss [5, 6]. In the distal oesophagus, leiomyomas may reach a large size [7]. In addition to basic examination methods, such as barium oesophagraphy, oesophago-gastro-duodenoscopy and CT, recently endosonography of the oesophagus is being indicated in all patients. EUS (endoscopic ultrasonography—EUS) has provided a major breakthrough for characterizing such masses by identifying the layer of origin and enabling guided-tissue acquisition for diagnostic studies, including immunohistochemistry. Endosonographically, leiomyomas are typically hypoechoic lesions with well-defined margins, although they have irregular margins and ulcerations on rare occasions. Preoperative biopsy is not generally recommended for a resectable lesion, and the patient is otherwise operable. EUS-guided biopsy has a variable diagnostic yield ranging from less than 20% to more than 90% according to the literature [8, 9]. However, a biopsy is preferred to confirm the diagnosis if advanced disease is suspected. Treatment options depend on the characteristics of the lesions. Small epithelial tumours are amenable to endoscopic removal, but bigger, lumen-obstructing masses often require surgical management. Nowadays, new endoscopic procedures are on hand in specialized centres offering a good curative option for patients with nonmalignant subepithelial tumours. Similarly to lower GI (gastrointestinal—GI) therapeutic endoscopy, EFTR (endoscopic full thickness resection—EFTR) and STER (submucosal tunnelling endoscopic resection—STER) have had promising clinical outcomes up to now, but long-term results are lacking. Most cases in the past have been managed with open resection, and certain cases call for a partial oesophagectomy with or without isoperistaltic jejunal interposition [10]. These patients require prolonged hospitalization and a long recovery time. Surgical enucleation using a minimally invasive approach represents the gold standard treatment in selected patients and is associated with less morbidity and a shorter hospital stay compared to thoracotomy. The majority of patients undergo a videothoracoscopic operation, but in specialized, high-volume upper GI institutions the laparoscopic-transhiatal procedure could be an alternative method for lower-third lesions. We report a successful operation of a duplex oesophageal leiomyoma using the laparoscopic-transhiatal approach.

## Case presentation

**Fig. 1:**
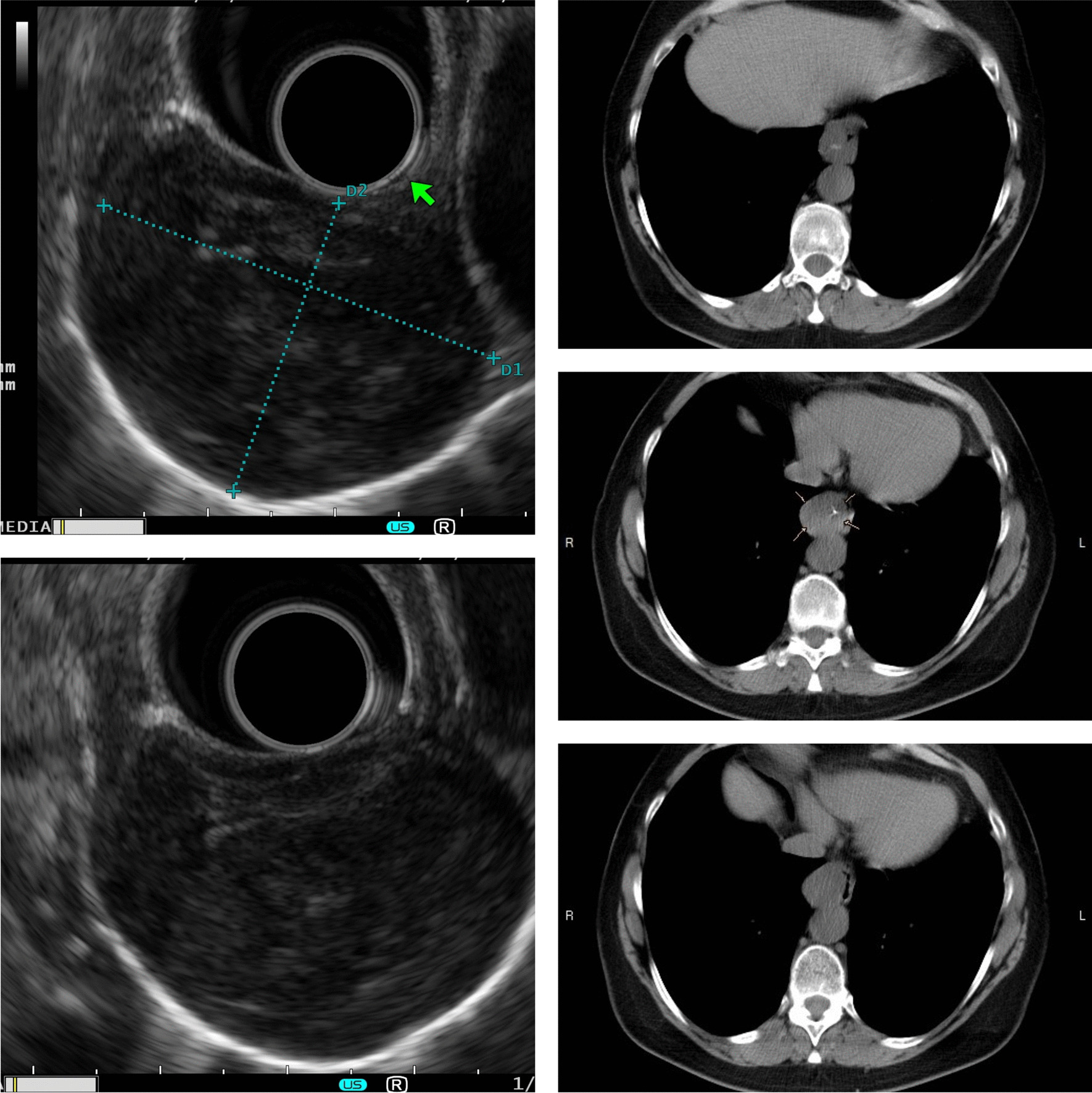
Computed tomography and endoscopic ultrasonography demonstrating wall thickening of the oesophagus causing stenotisation; photos were grouped together

An asymptomatic 60-year-old woman was incidentally diagnosed with a twin oesophageal mass, which was suspected due to mediastinal widening on a routine chest radiograph. A CT of the thorax discovered the lesions with eccentric oesophageal wall thickening in the distal third of the oesophagus, causing narrowing of the lumen (Fig. 1). Gastroscopy revealed an oesophageal submucosal protrusion with a smooth surface located 32 to 39 cm from the incisors, close to the Z line. The lesions were imaged as separate, 4 × 3 cm and 4 × 2 cm, inhomogeneous masses with some calcareous shadows arising from the muscularis propria on endoscopic ultrasound and were suspected to be leiomyomas because of their smooth surfaces and normal colours (Fig. 1). According to the NCCN (National Comprehensive Cancer Network—NCCN) Task Force Report, a biopsy may not be necessary if the tumour is easily resectable and preoperative therapy is not required [11]. Thus FNA (fine needle aspiration—FNA) of the lesions was not performed. The results of her physical examination were normal.

**Fig. 2:**
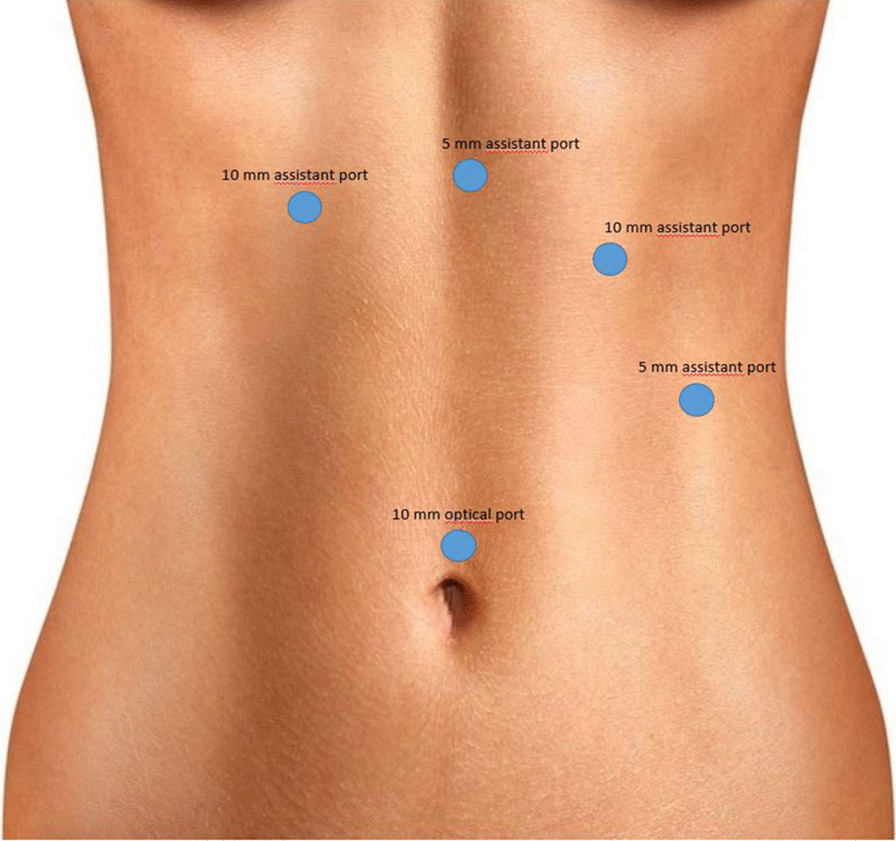
Port site positions

**Fig. 3:**
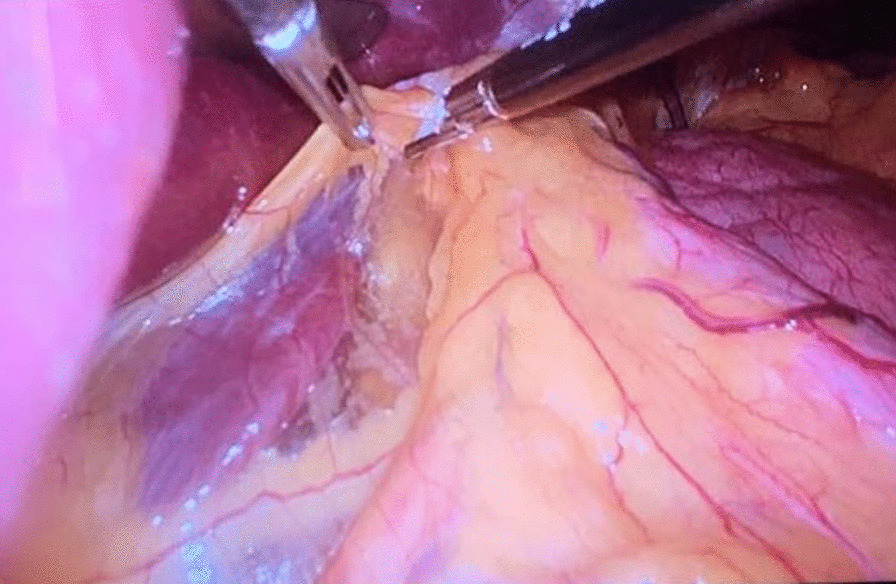
Dissection of the peritoneum

**Fig. 4:**
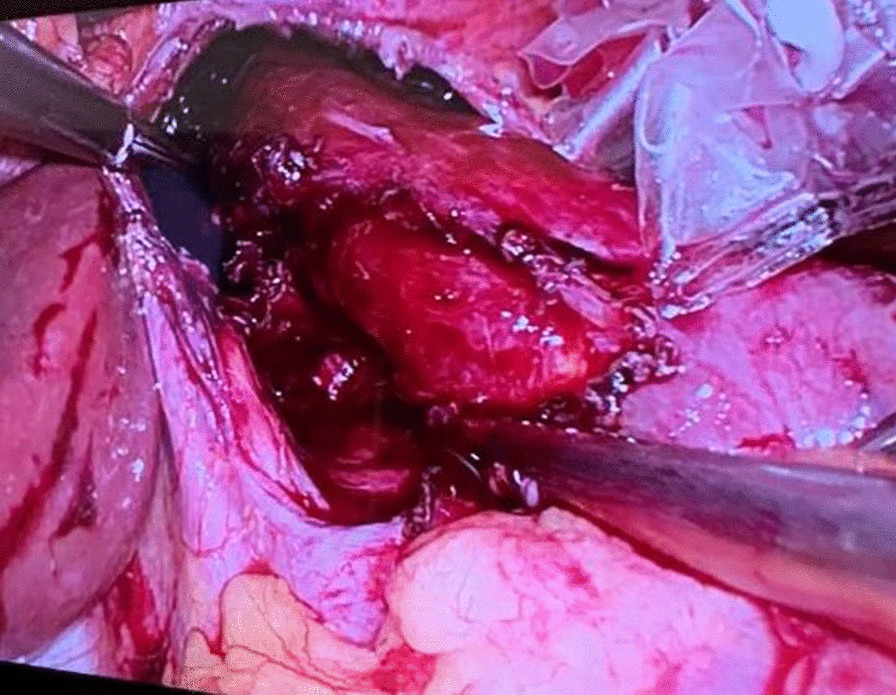
Checking mucosal integrity after myotomy

**Fig. 5:**
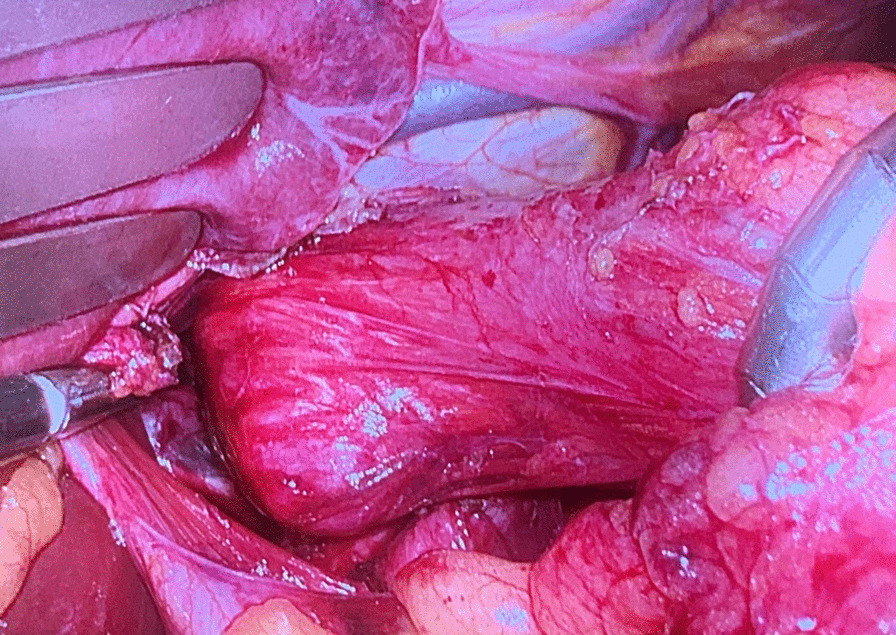
Intraoperative finding demonstrating a bulky mass above the GEJ

**Fig. 6:**
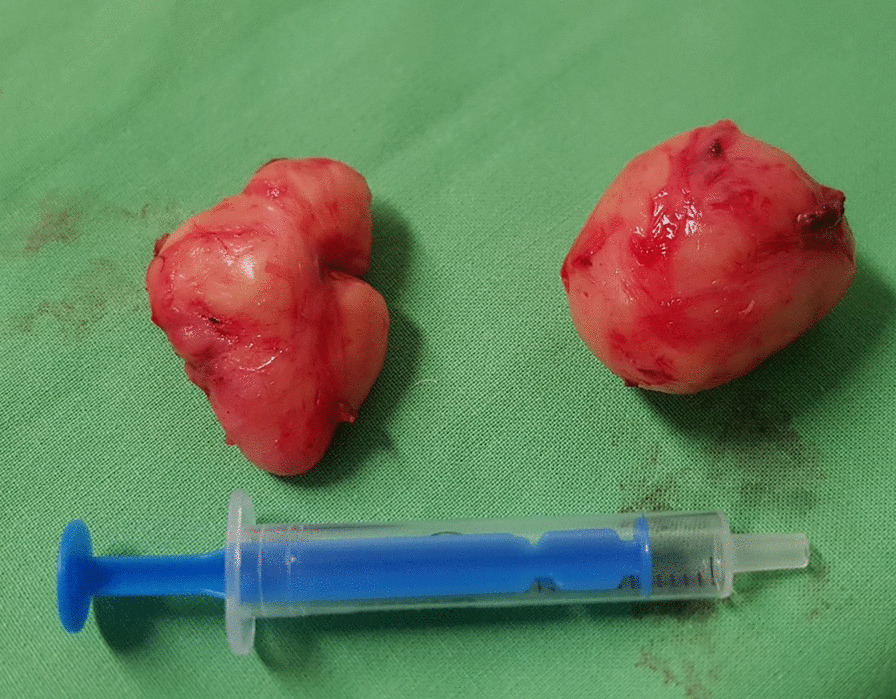
Two completely removed, separated, round leiomyomas

Before the scheduled operation, the patient had a history of a soft diet and received a prophylactic dose of LMWH (low-molecular-weight heparin—LMWH) a day before surgery. Third-generation cephalosporin and metronidazol were administered one hour before incision; then laparoscopic-transhiatal surgery was performed. The procedure was done under general anaesthesia with the patient in a semilithotomy, 25° reverse Trendelenburg position. Three ports were inserted in the abdominal cavity along the left costal arch, with one port placed in the epigastrium on the right side and one port, 10–12 mm in diameter, inserted directly above the umbilicus (Fig. 2). Slightly lower, intraabdominal pressure was set to 12 Hgmm to prevent pneumomediastinal side-effects. Laparoscopic exploration was done using a 30° camera. After retracting the left lobe of the liver, the peritoneum was divided up to the level of the median arcuate ligament with a Ligasure device (Valleylab, Boulder, CO, USA, Fig. 3). A nasogastric tube (size 16 Fr.) was placed into the oesophagus to identify its anatomical patterns and to avoid oesophageal side injuries. After incision of the hiatus (Pinotti’s manoeuvre), the lower third of the oesophagus was mobilised anteriorly; then transhiatal exploration revealed that the masses located right under each other (oral and aboral tumours) and orally had spread high to the middle third of the oesophagus. From the cardia side, approximately 3 cm above it, the muscularis of the oesophagus was cut in the expected line of myotomy to expose the capsule of the aborally located tumour. A well-defined solid tumour was revealed with an intact capsule and rich blood supply. The lower pole of the aboral tumour along the capsule was gradually isolated and removed using fine dissecting instruments and a high-energy cutting device. The incision was extended until it reached the upper edge of the orally located tumour, allowing for exploration of the oesophageal mucosa and gradual isolation of the tumour outside the mucosa until complete tumour resection was achieved. Technically, we opened the oesophageal muscle layer approximately 7–8 cm long, started 3 cm from the cardia and finished intramediastinally. The anterior vagal branch was under close monitoring and retracted during the entire operation. The integrity of the mucosal membrane of the oesophagus was checked with intraoperative endoscopy; the leak test during air insufflation was negative (Fig. 4). The oesophageal muscular wall was repaired by absorbable 3/0 monofilament sutures. Fundoplication was not added because the localisation of the duplex tumour was at least 3 cm above the cardia so the natural antireflux mechanisms were not compromised during surgery. On the other hand, the wrapped segment would have formed more distally from the sutured oesophageal wall. The specimens were removed in an endobag. The operation took approximately 120 min to complete (Fig. 5). The tumours were approximately 38 × 29 × 27 mm and 37 × 20 × 30 mm in diameter and tough in quality, with a well-defined round shape (Fig. 6). After the operation, the patient underwent water fasting, gastrointestinal decompression and acid suppression. Contrast radiography of the oesophagus on the fifth postoperative day showed intact smooth oesophageal mucosa with no obvious leakage, so the nasogastric tube was removed and the patient began to consume liquid food. She was discharged on the 10th postoperative day. She has been followed up for six months with a normal swallowing test, adequate oesophageal function and no complaints observed. A pathological examination confirmed the diagnosis of leiomyoma in both lesions. Immunohistochemistry resulted negative for DOG1 (discovered on GIST1—DOG1) and C-kit (CD117) and positive for SMA (smooth muscle actin—SMA) and desmin. The mitotic rate (Ki-67 index) was low. The specimen assessment showed complete removal of the two lesions, with no damage to the capsulae seen.

## Discussion and conclusions

Surgical excision (enucleation) is recommended for symptomatic leiomyomas and those greater than 5 cm. Traditionally, tumours of the middle third of the oesophagus are approached using a right thoracotomy; tumours in the distal third of the oesophagus are resected through a left thoracotomy, with all its associated morbidity [12]. While the open surgical technique is still the mainstay of therapy for leiomyomas, combined oesophagoscopy and video-assisted resection (thoracoscopy and laparoscopy) are being increasingly performed [13–16]. Because of their well-known advantages, thoracoscopic, laparoscopic, endoscopic and hybrid procedures have replaced conventional surgery in specialized centres. Laparoscopic-transhiatal enucleation is preferred for lesions in the distal third and near the GEJ (gastroesophageal junction—GEJ) because it provides perfect exposure of the lower mediastinum and the upper abdomen, allowing us to perform an antireflux repair (anterior—Dor or posterior—Toupet fundoplication). For upper- and middle-third leiomyomas, the left-sided VATS (video-assisted thoracoscopic surgery—VATS) approach is accepted, as it provides better exposure and access to the entire oesophagus [17, 18]. Jesic et al. and Smith et al. support the transhiatal approach for lower oesophageal tumours [19, 20].

To the best of our knowledge, this is the first reported case of complete laparoscopic transhiatal enucleation of twin oesophageal leiomyomas. There has been no reported case of duplex oesophageal leiomyomas removed laparoscopically or thoracoscopically to date. If located in the mediastinum, videothoracoscopic enucleation is considered the gold standard approach in patients with small-to-medium oesophageal leiomyomas [4]. We have shown that even a more orally located leiomyoma can be safely resected through a laparoscopic approach in experienced hands. The pathological examination showed the intact capsulae of the tumours, and we have no difficulty preserving the vagal nerve and the mucous membrane of the oesophagus. We expected this because the patient did not receive a preoperative biopsy, which would have made the operation challenging. If an oesophageal leiomyoma is suspected, many authors do not recommend diagnostic punctures of the lesion. When the mucosa appears intact on endoscopy, preoperative diagnostic punctures can cause obliteration of the layers of the oesophageal wall, resulting in difficulties during surgical enucleation and increased risk of mucosal perforation and postoperative complications [21, 22]. In our patient, the tumours were leiomyomas of the lower third, which is the most common location, but duplication is extremely rare. Endoscopic ultrasonography is the preferred modality in the evaluation process and should be employed early. In clinical practice, surgical intervention or endoscopic resection is usually recommended to prevent excessive growth and related complications [5].

Introduced by pioneers from the Far East, rapid adoption of advanced endoscopic techniques has been observed in minimally invasive treatment for oesophageal subepithelial tumours. Using the principles of POEM (per-oral endoscopic myotomy—POEM), the EFTR method has spread increasingly worldwide. During EFTR, operators cut the mucosa at the level of the tumour; submucosal dissection is then performed to free the tumour. Muscle fibre dissection is carried out along the capsule to remove the lesion; then the defect is closed. There is another form of endoscopic resection called STER, which was first described by the POEM innovators from China and Japan [23, 24]. STER begins with a mucosal incision a few centimeters above the tumour, continues with tunnelling around the tumour while separating it from the mucosa covering it and the muscle fibres around it until it is excavated from attachments to the muscularis. The mucosal damage is significantly less in contrast to EFTR. STER has the great advantage of approaching tumours in critical localisation (GEJ and gastric antrum), where surgical resection is technically challenging [25]. Unlike EFTR, the benefit of STER is the closing method compared to a full-thickness defect. The vast majority of studies are retrospective data with limited follow-up. A recent meta-analysis on STER demonstrated an en bloc resection rate of 94.9%, with overall gas-related and inflammation-related adverse events rates of 21.5% and 8.4%, respectively, a postinterventional bleeding rate of 2.2%, and length of hospital stay of a median of 3.8 days [26]. Wang et al. published excellent outcomes with the STER procedure by successfully removing multiple leiomyomas with a mean diameter of 15 mm in 12 patients [27]. In a prospective, comparative study with 66 patients, STER and VATS were investigated. The authors found a shorter procedure time (44.5 vs. 106.5 min), lower cost ($4499 vs. $5137), less decrease in haemoglobin level (0.16 vs. 1.47 g/dL) and lower postoperative pain scores in the STER group and comparable perioperative clinical outcomes (complete resection rate, hospital times and adverse events) between the two groups apart from a lower en bloc resection rate for STER for subepithelial tumours of ≥ 2 cm (71.4% vs. 100%) [28]. STER seems to be a good alternative to surgical resection in patients with small subepithelial lesions, but even dealing with a large size can be safe and feasible [29]. On the other hand, EFTR and STER have some contraindications, including severe comorbidity, sign of advanced-stage tumour and adjacency to large extra-luminal vessels. In planning the endoscopic treatment, there are no fixed criteria on the size and location of the lesions; it rather depends on the expertise of the operator. However, tumours bigger than 3–4 cm in the shortest diameter may cause difficulties retracting from the mouth, and tumours over 5 cm in the longest diameter have a significantly higher risk of malignant potential, with surgery thus offering a better outcome. If multiple tumours are located at the same level of the oesophagus, endoscopic techniques may be challenging and carry significant risk of complication even in experienced hands. Due to the short follow-up, R1 (microscopic residual disease) cases, and possible early recurrences and residual tumours, concerns developed among the authors and time will be needed to elucidate the role of these novel endoscopic techniques. EFTR and STER, these two frontier endoscopic tools, are highly promising, but long-term data are missing and prospective randomised studies are lacking.

Robotic surgery could be another highly sophisticated treatment option in some referral centres. The procedure is safe, provides comparable outcomes to thoracoscopy and offers better ergonomics to well-trained surgeons, but only in very few, highly selected patients [30, 31]. Cerfolio et al. reported four successful leiomyoma resections using a robotic platform, demonstrating the biggest series worldwide in this area [32].

Recently, Asti et al. reported excellent perioperative and long-term results with a minimally invasive approach (thoracoscopic, laparoscopic and endoscopic) in 35 patients with leiomyomas near the GEJ. They performed an additional semifundoplication, eliminating dependence on the PPI (proton pump inhibitor—PPI) in the nonendoscopic group; after a median of 49 months, no recurrences and good swallowing function were documented [33]. Obuchi et al. performed laparoscopic-transhiatal enucleation in three cases with a 3.9 cm mean tumour size and no major morbidity or mortality. At a mean follow-up period of 60.1 months, recurrence was not demonstrated [34].

We also prefer the laparoscopic transhiatal approach for benign lower-third lesions, and its feasibility was demonstrated by our previous study with the transhiatal management of epiphrenic oesophageal diverticula [35]. In experienced hands, the lower oesophagus can be mobilised to the mediastinum without pleura injury. In addition, the laparoscopic approach can be advantageous in patients with possible intrathoracic adhesions due to a history of previous mediastinal inflammation and in patients with reduced lung capacity. When the operation is combined with intraoperative oesophagoscopy, several advantages are on hand: first, localising the tumour is easier; second, checking the mucous membrane after enucleation can minimize postoperative complications [36]. If the tumour is located at least 3 cm above the GEJ, GERD (gastroesophageal reflux disease—GERD)-associated symptoms are lacking and the oesophageal wall can be reconstructed successfully; there is therefore no need for semifundoplication [37, 38]. Laparoscopic transhiatal enucleation of lower oesophageal leiomyomas and other benign tumours is a safe, fast and effective operation, and offers a good therapeutic alternative to VATS.

## Data Availability

This case report contains clinical data from the electronic medical record at the Department of Surgery, University of Szeged. Additional information is available from the corresponding author on reasonable request from the editor.

## Abbreviations

CT: Computed tomography
EUS: Endoscopic ultrasonography
GI: Gastrointestinal
EFTR: Endoscopic full thickness resection
STER: Submucosal tunnelling endoscopic resection
NCCN: National Comprehensive Cancer Network
FNA: Fine needle aspiration
LMWH: Low-molecular-weight heparin
DOG1: Discovered on GIST1
SMA: Smooth muscle actin
GEJ: Gastroesophageal junction
VATS: Video-assisted thoracoscopic surgery
POEM: Per-oral endoscopic myotomy
GIST: Gastrointestinal stromal tumour
PPI: Proton pump inhibitor
GERD: Gastroesophageal reflux disease
